# Supplementary material for: Analysis of human acetylation stoichiometry defines mechanistic constraints on protein regulation
Source: Nat Commun. 2019 Mar 5;10:1055. doi: 10.1038/s41467-019-09024-0 (PMC6401094; doi:10.1038/s41467-019-09024-0)
Supplement: Supplementary file 1 — Supplementary Information [file 41467_2019_9024_MOESM1_ESM.pdf]

Supplementary Information

Analysis of human acetylation stoichiometry defines mechanistic constraints on protein regulation

Bogi K. Hansen[1], Rajat Gupta[1], Linda Baldus[2,3], David Lyon[1], Takeo Narita[1], Michael Lammers[2,3], Chunaram Choudhary[1,*] and Brian T. Weinert[1,*]

[1]Department of Proteomics, The Novo Nordisk Foundation Center for Protein Research, Faculty of Health and Medical Sciences, University of Copenhagen, Blegdamsvej 3B, DK-2200 Copenhagen, Denmark.

[2]Institute of Biochemistry, Synthetic and Structural Biochemistry, University of Greifswald, Greifswald, Germany

[3]Institute for Genetics and Cologne Excellence Cluster on Cellular Stress Responses in Aging-Associated Diseases, CECAD, University of Cologne, Joseph-Stelzmann-Str. 26, 50931, Cologne, Germany.

[*]Correspondence to: brian.weinert@gmail.com and chuna.choudhary@cpr.ku.dk

Supplementary Figure 1

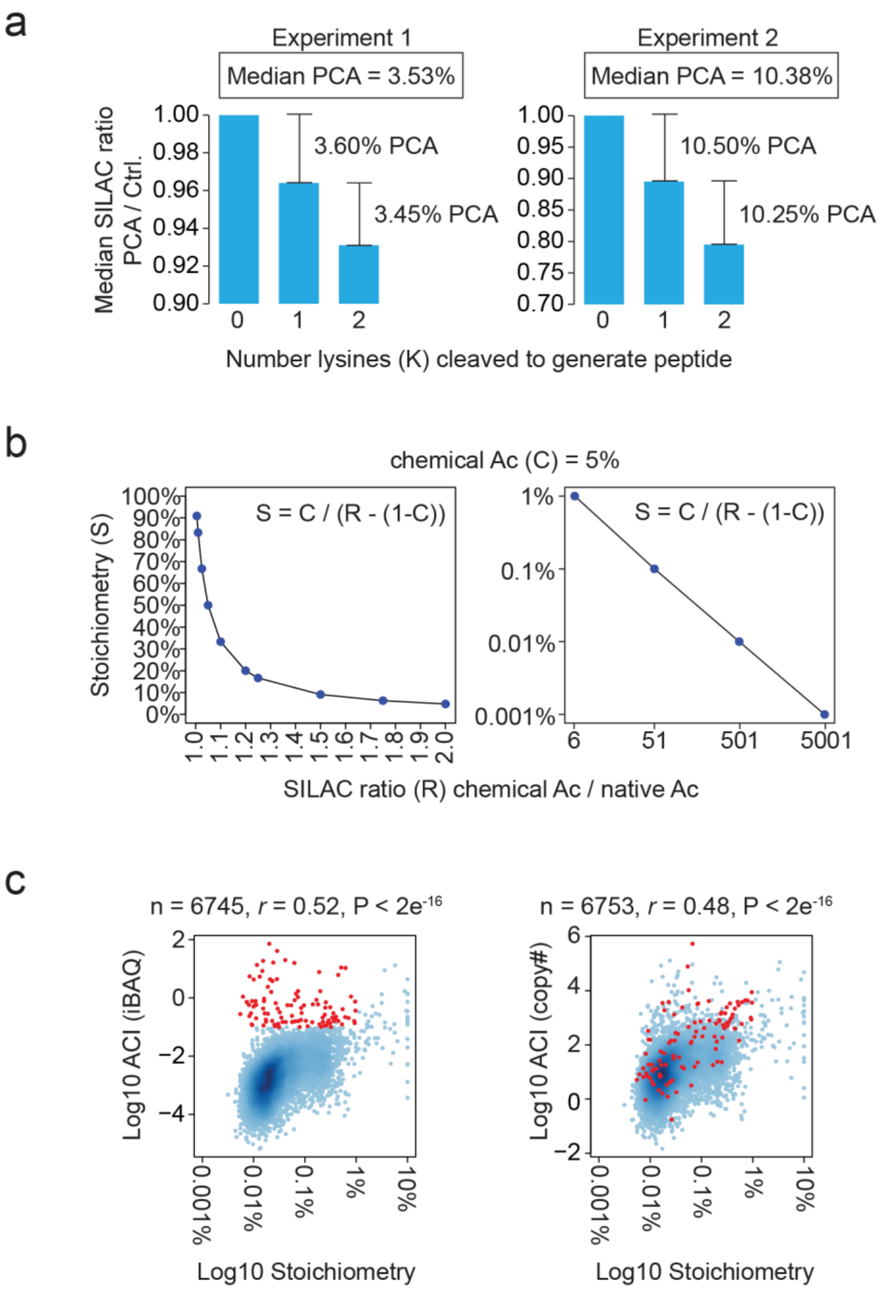

Supplementary Figure 1

Measuring acetylation stoichiometry (**a**) Measuring the degree of partial chemical acetylation (PCA). The column charts show the median SILAC ratios for the indicated classes of peptides. The number of peptides analyzed is K0 (4,316), K1(4,713), K2(3,635) for experiment 1 and K0 (900), K1(5,166), K2(9,304) for experiment 2. The degree of PCA is equal to the median reduced cleavage at lysine residues, as shown in brackets. (**b**) Model showing the relationship between SILAC ratio and stoichiometry when PCA equals 5%. The equation used to calculate stoichiometry (S) based on the degree PCA (C) and the SILAC ratio (R) chemical acetylated / native acetylated peptide, is shown. (**c**) The scatterplots show the relationship between abundance corrected intensity (ACI) and acetylation stoichiometry. ACI was calculated using either iBAQ-based abundance estimates or by using copy numbers as determined by [1]. Outlier data points using iBAQ-based ACI are colored red in both plots and are mostly not outliers when using copy-number-based ACI. The number of peptides (n), Pearson's correlation (*r*), and P-value (P) of correlation are shown. Source data are provided as a Source Data file.

Supplementary Figure 2

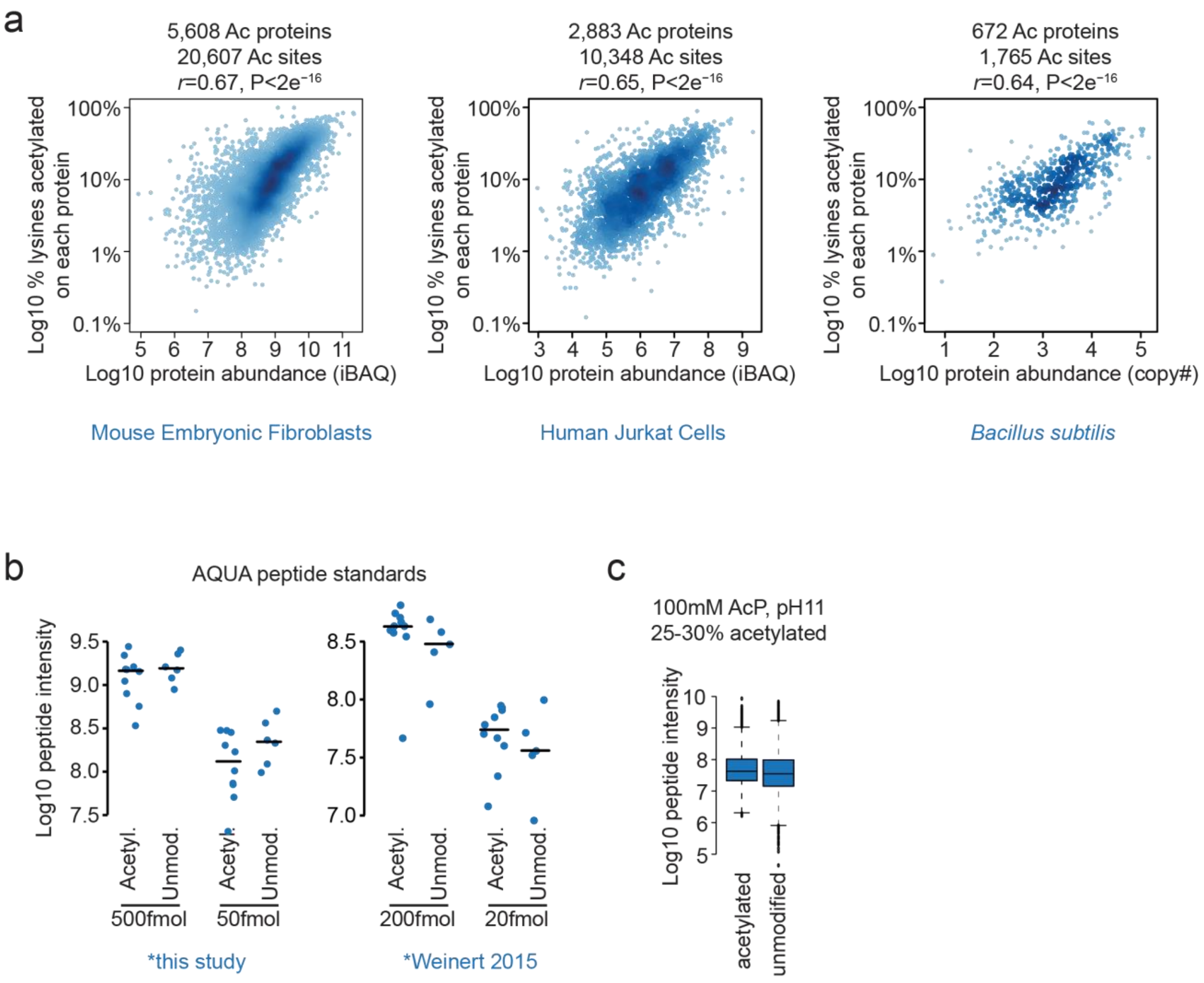

Supplementary Figure 2

Copy number limits the detection acetylated peptides (**A**) Measuring the degree of partial chemical acetylation (PCA). The column charts show the median SILAC ratios for the indicated classes of peptides. The degree of PCA is equal to the reduced cleavage at lysine residues, as shown. Data used to generate the plots is from: mouse embryonic fibroblasts [2], Jurkat [3], and *Bacillus subtilis* [4]. (**B**) The category scatterplots show the intensity of the indicated quantities of acetylated (acetyl.) and unmodified (unmod.) AQUA peptide standards from this study and from our previous analysis of acetylation stoichiometry in mouse liver tissue [5]. The black line indicates the median intensity. (**C**) The boxplots show the distributions of acetylated (4,373) and unmodified (6,777) peptide intensities in a sample that was 25-30% chemically acetylated by treatment with acetyl-phosphate at high pH [5]. The box shows the middle 50% of the data points (the interquartile range (IQR)) and the line indicates the median value, whisker ends extend to 1.5x IQR, outliers are shown. Source data are provided as a Source Data file.

Supplementary Figure 3

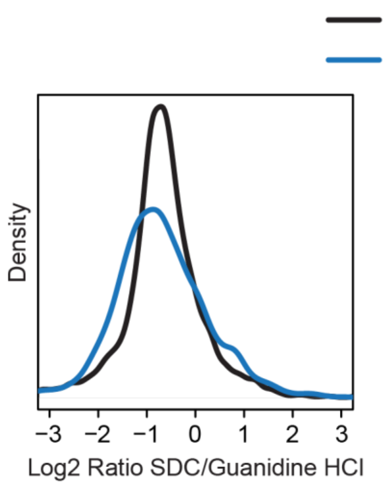

Supplementary Figure 3

The method of cell lysis and protein extraction has no substantial impact on protein acetylation. SILAC Heavy labeled HeLa cells were lysed in 2% sodium deoxycholate (SDC) in 50mM Hepes pH8 and SILAC Light labeled HeLa cells were lysed in either 8M Guanidine HCl, 50mM Hepes pH8 or in modified RIPA buffer followed by acetone precipitation as used in this study and described in the Materials and Methods. The density plots show the not-normalized distributions of SILAC ratios from unmodified and acetylated peptides when comparing the SDC lysis to Guanidine HCl lysis, or SDC lysis to RIPA lysis. Source data are provided as a Source Data file.

Supplementary References

1. Kulak NA, Pichler G, Paron I, Nagaraj N, Mann M. Minimal, encapsulated proteomic-sample processing applied to copy-number estimation in eukaryotic cells. *Nature methods* **11**, 319-324 (2014).

2. Weinert BT*, et al.* Time-Resolved Analysis Reveals Rapid Dynamics and Broad Scope of the CBP/p300 Acetylome. *Cell* **174**, 231-244 e212 (2018).

3. Svinkina T*, et al.* Deep, Quantitative Coverage of the Lysine Acetylome Using Novel Anti-acetyl-lysine Antibodies and an Optimized Proteomic Workflow. *Mol Cell Proteomics* **14**, 2429-2440 (2015).

4. Carabetta VJ, Greco TM, Tanner AW, Cristea IM, Dubnau D. Temporal Regulation of the Bacillus subtilis Acetylome and Evidence for a Role of MreB Acetylation in Cell Wall Growth. *mSystems* **1**, (2016).

5. Weinert BT, Moustafa T, Iesmantavicius V, Zechner R, Choudhary C. Analysis of acetylation stoichiometry suggests that SIRT3 repairs nonenzymatic acetylation lesions. *The EMBO journal* **34**, 2620-2632 (2015).
