# Supplementary material for: Analysis of human acetylation stoichiometry defines mechanistic constraints on protein regulation
Source: Nat Commun. 2019 Mar 5;10:1055. doi: 10.1038/s41467-019-09024-0 (PMC6401094; doi:10.1038/s41467-019-09024-0)
Supplement: Supplementary file 3 — Description of Additional Supplementary Files [file 41467_2019_9024_MOESM3_ESM.docx]

**Description of Supplementary Files**

**File Name:** Supplementary Data 1

**Description:** (a) Acetylated peptide SILAC ratios and stoichiometry calculations. (b) Acetylated peptide stoichiometry measurements using known quantities of AQUA peptide standards. (c) Site-level acetylation stoichiometry data. (d) UniProt Keyword enrichment analysis comparing proteins with >0.23% stoichiometry and cysteine residues in the -2, -3, or -4 positions to all proteins with stoichiometry measurements.
