# Supplementary material for: Analysis of human acetylation stoichiometry defines mechanistic constraints on protein regulation
Source: Nat Commun. 2019 Mar 5;10:1055. doi: 10.1038/s41467-019-09024-0 (PMC6401094; doi:10.1038/s41467-019-09024-0)
Supplement: Supplementary file 5 — Reporting Summary [file 41467_2019_9024_MOESM5_ESM.pdf]

nature research

Corresponding author(s): Brian Tate Weinert
Chunaram Choudhary

Reporting Summary

Nature Research wishes to improve the reproducibility of the work that we publish. This form provides structure for consistency and transparency in reporting. For further information on Nature Research policies, see Authors & Referees and the Editorial Policy Checklist.

Statistical parameters

When statistical analyses are reported, confirm that the following items are present in the relevant location (e.g. figure legend, table legend, main text, or Methods section).

n/a	Confirmed	
☐	☒	The exact sample size (*n*) for each experimental group/condition, given as a discrete number and unit of measurement
☐	☒	An indication of whether measurements were taken from distinct samples or whether the same sample was measured repeatedly
☐	☒	The statistical test(s) used AND whether they are one- or two-sided<br>*Only common tests should be described solely by name; describe more complex techniques in the Methods section.*
☒	☐	A description of all covariates tested
☒	☐	A description of any assumptions or corrections, such as tests of normality and adjustment for multiple comparisons
☐	☒	A full description of the statistics including central tendency (e.g. means) or other basic estimates (e.g. regression coefficient) AND variation (e.g. standard deviation) or associated estimates of uncertainty (e.g. confidence intervals)
☒	☐	For null hypothesis testing, the test statistic (e.g. *F*, *t*, *r*) with confidence intervals, effect sizes, degrees of freedom and *P* value noted<br>*Give P values as exact values whenever suitable.*
☒	☐	For Bayesian analysis, information on the choice of priors and Markov chain Monte Carlo settings
☒	☐	For hierarchical and complex designs, identification of the appropriate level for tests and full reporting of outcomes
☐	☒	Estimates of effect sizes (e.g. Cohen's *d*, Pearson's *r*), indicating how they were calculated
☐	☒	Clearly defined error bars<br>*State explicitly what error bars represent (e.g. SD, SE, CI)*

Our web collection on statistics for biologists may be useful.

Software and code

Policy information about availability of computer code

Data collection	Raw MS data was analyzed using MaxQuant (developer version 1.5.5.4) to search the UniProt human FASTA (downloaded July 6, 2015).
Data analysis	Data were analyzed using R studio (Version 1.1.383) running R version 3.4.3. Calculation of acetylation stoichiometry is described in detail in the methods section.

For manuscripts utilizing custom algorithms or software that are central to the research but not yet described in published literature, software must be made available to editors/reviewers upon request. We strongly encourage code deposition in a community repository (e.g. GitHub). See the Nature Research guidelines for submitting code & software for further information.

Data

Policy information about availability of data

All manuscripts must include a data availability statement. This statement should provide the following information, where applicable:

- Accession codes, unique identifiers, or web links for publicly available datasets
- A list of figures that have associated raw data
- A description of any restrictions on data availability

The raw mass spectrometry data have been deposited to the ProteomeXchange Consortium via the PRIDE (https://www.ebi.ac.uk/pride) partner repository with the dataset identifier PXD009994 (Figures 1-4).

April 2018

Field-specific reporting

Please select the best fit for your research. If you are not sure, read the appropriate sections before making your selection.

☒ Life sciences ☐ Behavioural & social sciences ☐ Ecological, evolutionary & environmental sciences

For a reference copy of the document with all sections, see nature.com/authors/policies/ReportingSummary-flat.pdf

Life sciences study design

All studies must disclose on these points even when the disclosure is negative.

Sample size	Sample size was not determined since we were not examining statistically significant differences between two groups.
Data exclusions	Doubly acetylated peptides were not used to calculate acetylation stoichiometry. Peptides that were only measured at one dilution point were excluded because we could not verify the accuracy of these measurements. Peptides with SILAC ratios that differed by more than two-fold between measurements made at two different dilutions were not confidently quantified and were excluded from analysis.
Replication	Stoichiometry was measured in two biological replicates from cells with inverted SILAC ratios (in the first experiment SILAC heavy peptides were chemically acetylated, in the second experiment SILAC light peptides were chemically acetylated). Each experiment was measured at three different dilutions of chemically acetylated peptides (three technical replicates), and only peptides whose measured SILAC ratios agreed between two or more technical replicates (at different dilutions) were used to calculate stoichiometry.
Randomization	SILAC labeling was switched between biological replicate experiments to control for biased introduced by SILAC labeling.
Blinding	Blinding was not performed as there is no control group, we are measuring a physical parameter.

Reporting for specific materials, systems and methods

Materials & experimental systems

n/a	Involved in the study
☒	☐ Unique biological materials
☒	☐ Antibodies
☐	☒ Eukaryotic cell lines
☒	☐ Palaeontology
☒	☐ Animals and other organisms
☒	☐ Human research participants

Methods

n/a	Involved in the study
☒	☐ ChIP-seq
☒	☐ Flow cytometry
☒	☐ MRI-based neuroimaging

Eukaryotic cell lines

Policy information about cell lines

Cell line source(s)	HeLa (ATCC: CCL-2) cells, source ATCC
Authentication	Not authenticated
Mycoplasma contamination	Tested negative for mycoplasma
Commonly misidentified lines (See ICLAC register)	*Name any commonly misidentified cell lines used in the study and provide a rationale for their use.*
